# Innovative pathways in revascularization for non-ST elevation acute coronary syndrome

**DOI:** 10.1016/j.xjon.2024.07.017

**Published:** 2024-08-02

**Authors:** FNU Venjhraj, Vikram Singh, Ashvin Kumar, Aiman Salam Shaikh

**Affiliations:** a Internal Medicine, Shaheed Mohtarma Benazir Bhutto Medical College Lyari, Karachi, Pakistan; b Institute of Medical Technology, Jinnah Sindh Medical University, Karachi, Pakistan

To the Editor:

**Figure:**
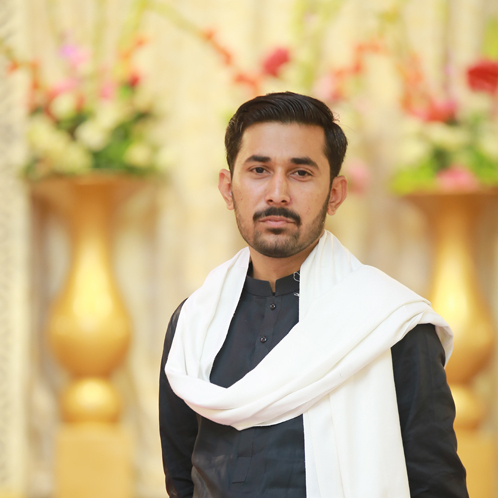

We have read the article by Ram and colleagues,^1^ “Outcomes of different revascularization strategies among patients presenting with acute coronary syndromes without ST elevation.” It is important for the reader to recognize the author’s efforts in tackling such a sensitive subject; however, we cannot resist adding a few things to these articles to improve them and broaden the reader’s comprehension.

Patients with severe acute coronary syndrome have tortuous and calcified coronary arteries, making angiography less reliable. Because of this, it can be challenging to diagnose myocardial ischemia in patients undergoing transcatheter aortic valve replacement. There is little association between intracoronary physiology and angiography, particularly in the left anterior descending artery region.^2^

Forensic pathologists determine the cause of sudden deaths using police reports, medical records, autopsies, and additional analyses. Standard protocols guide the process, with specialist consultations when needed. Causes are classified using International Classification of Diseases 10 codes, and autopsies involve mandatory histologic examinations. In unclear cases, toxicology tests for drugs and alcohol are conducted. Detailed cardiac investigations are performed during autopsies, measuring heart weight, dissecting and examining the myocardium and arteries, and analyzing tissue samples. Acute coronary complications, such as fresh thrombi or critical stenosis, define ischemic sudden death. Hearts exceeding expected weight and containing enlarged muscle cells are classified as having left ventricular hypertrophy.^3^

After isolated coronary artery bypass grafting (CABG), 30% of patients develop atrial fibrillation. According to the 2020 Canadian Cardiovascular Society Comprehensive Guidelines, each patient should receive customized treatment that includes rate or rhythm management. Patients may be released from the hospital on anticoagulation, with β-blockers, amiodarone, or both—specifically, warfarin—to decrease stroke risk. After CABG, most patients experience self-limiting, new-onset atrial fibrillation and return to normal sinus rhythm within 6 to 12 weeks. After 3 months, the primary care physician should reassess the patient's anticoagulant therapy; if the patient is experiencing atrial fibrillation solely after heart surgery and has no arrhythmias, stopping anticoagulation may be an option.^4^

According to Ram and colleagues, the periprocedural risk often is higher worldwide in registries than in randomized groups. Compared to percutaneous intervention (PCI), CABG consistently performs better in subgroups whose myocardial infarction rates are lower in low-risk randomized populations. Risk-adjusted registry comparisons favor CABG for long-term results, which may be explained by patients’ increased myocardial infarction risk in everyday practice compared to randomized trials. All studies that have published rates showed a lower rate of myocardial infarctions in the CABG population. Furthermore, surgical denervation may arise from the distal anastomosis tissue manipulation performed during CABG, which could explain why CABG is superior to PCI in terms of survival and possibly long-term quality of life. The most startling conclusion is that, based on the data from observational studies, there was no difference in periprocedural mortality between PCI and CABG. In conclusion, data from randomized populations and routine use of CABG seem to indicate that CABG is far less harmful than anticipated.^5^

## Conflict of Interest Statement

The authors reported no conflicts of interest.

The *Journal* policy requires editors and reviewers to disclose conflicts of interest and to decline handling or reviewing manuscripts for which they may have a conflict of interest. The editors and reviewers of this article have no conflicts of interest.
